# Randomized controlled trial of medium cut-off versus high-flux dialyzers on quality of life outcomes in maintenance hemodialysis patients

**DOI:** 10.1038/s41598-020-64622-z

**Published:** 2020-05-08

**Authors:** Jeong-Hoon Lim, Yeongwoo Park, Ju-Min Yook, Soon-Youn Choi, Hee-Yeon Jung, Ji-Young Choi, Sun-Hee Park, Chan-Duck Kim, Yong-Lim Kim, Jang-Hee Cho

**Affiliations:** 1 Department of Internal Medicine, School of Medicine, Kyungpook National University, Kyungpook National University Hospital, Daegu, South Korea; 2 Department of Statistics, Kyungpook National University, Daegu, South Korea

**Keywords:** Haemodialysis, End-stage renal disease

## Abstract

Medium cut-off (MCO) dialyzers help remove larger middle molecules associated with symptoms related to the accumulation of uremic retention solutes. We investigated the effect of an MCO dialyzer on the improvement of quality of life (QOL) in maintenance hemodialysis (HD) patients. Forty-nine HD patients with high-flux dialysis were randomly assigned to either an MCO (Theranova 400, Baxter) or a high-flux (FX CorDiax 80 or 60, Fresenius Medical Care) dialyzer and completed the study. QOL was assessed at baseline and after 12 weeks of treatment using the Kidney Disease Quality of Life Short Form-36, and pruritus was assessed using a questionnaire and visual analog scale. The reduction ratios of middle molecules were also evaluated. Laboratory markers, including serum albumin, did not differ between the two groups after 12 weeks. Removals of kappa and lambda free light chains were greater for MCO dialyzer than high-flux dialyzer. The MCO group had higher scores than the high-flux group in the domains of physical functioning and physical role (75.2 ± 20.8 vs. 59.8 ± 30.1, *P* = 0.042; 61.5 ± 37.6 vs. 39.0 ± 39.6, *P* = 0.047, respectively), and the MCO group had lower mean scores for morning pruritus distribution and the frequency of scratching during sleep (1.29 ± 0.46 vs. 1.64 ± 0.64, *P* = 0.034; 0.25 ± 0.53 vs. 1.00 ± 1.47, *P* = 0.023, respectively). MCO dialyzers may improve patient-reported outcomes, particularly the physical components of QOL and uremic pruritus, in patients with high-flux dialyzers.

## Introduction

Patients on maintenance dialysis suffer variable symptoms such as fatigue, generalized weakness, and pruritus. These subjective conditions are assumed to be related to middle molecules that are not cleared by conventional hemodialysis (HD)^1^. Middle molecules have molecular weights (MWs) ranging between 500 and 60,000 daltons, and their size is a barrier to removal with dialyzers^2,3^. The accumulation of middle molecules is associated with specific complications such as amyloidosis, inflammatory reactions, oxidative stress, and endothelial dysfunction^3–5^. Consequently, middle molecules contribute to morbidity and mortality and poor quality of life (QOL) in end-stage renal disease (ESRD) patients^6,7^.

High-flux dialysis, which has benefits for middle molecule clearance, has not displayed a clear mortality advantage compared with low-flux dialysis; the survival benefits of high-flux dialysis were only observed in patients with hypoalbuminemia and diabetes^8,9^. Another dialytic modality, hemodiafiltration (HDF) with increased convection, has a higher efficiency for reducing middle-sized solutes than high-flux dialysis^10,11^. Large randomized trials comparing HDF and high-flux dialysis have shown conflicting patient outcome results^12–15^; however, recent results have consistently shown that HDF with a high convection volume improves patient survival^16–18^.

Medium cut-off (MCO) dialyzers are characterized by a more even distribution of larger pore sizes and a higher number of pores. MCO dialyzers have a higher permeability and have increased convective transport because of their significant amount of internal filtration^19^, so they can potentially improve the removal of middle molecules compared with high-flux dialyzers and even compared with HDF^20^. However, clinical data about the effect of MCO dialyzers on patient-reported outcomes are lacking. The aim of this study was to investigate whether MCO dialyzers could improve QOL in maintenance HD patients treated with a high-flux dialyzer. We also evaluated the effect of MCO dialyzers on pre-dialysis plasma concentrations and the removal of middle molecules.

## Results

### Patient characteristics

**Figure 1:**
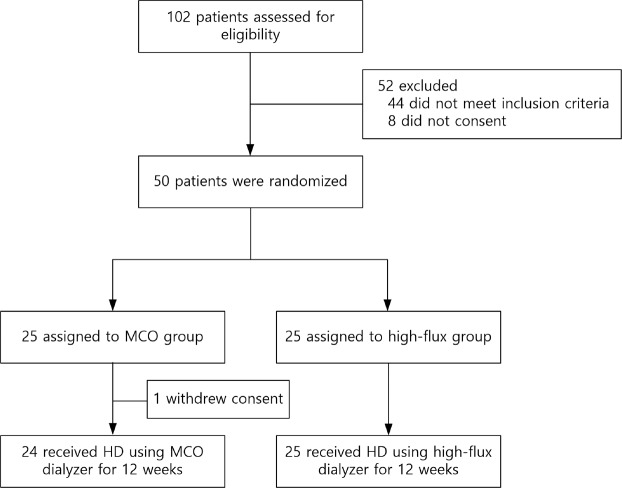
Flow diagram of the study protocol. Abbreviations: MCO, medium cut-off; HD, hemodialysis.

A total of 50 patients were enrolled in this study, and one patient withdrew consent (Fig. 1). Among the 49 patients who completed the study, 24 were in the MCO group and 25 were in the high-flux group. The baseline characteristics did not differ between the two groups (Table 1). The mean age of patients in the MCO group was 62.2 ± 13.7 years, and 75.0% were men. The high-flux group patients had a similar mean age and percentage of male participants (63.8 ± 15.2 years and 60.0%, respectively). Body mass index, dry weight, daily urine volume, vascular access, baseline dialyzer, and dialysis vintage did not differ between the groups.

**Table 1:** Baseline characteristics.

	MCO (*n* = 24)	High-flux (*n* = 25)	*P*
Age, y	62.2 ± 13.7	63.8 ± 15.2	0.687
Male gender, n (%)	18 (75.0)	15 (60.0)	0.364
Asian race, n (%)	24 (100.0)	25 (100.0)	1.000
Body mass index, kg/m^2^	22.0 ± 2.6	21.8 ± 3.8	0.812
Dry weight (kg)	61.0 ± 7.6	56.6 ± 9.9	0.089
Urine volume (mL/day)	75.8 ± 216.9	147.2 ± 366.3	0.413
Primary renal disease, n (%)			0.458
Diabetes	11 (45.8)	12 (48.0)	
Hypertension	3 (12.5)	5 (20.0)	
Glomerulonephritis	9 (37.5)	5 (20.0)	
Others	1 (4.2)	3 (12.0)	
Comorbid conditions, n (%)			
Hypertension	19 (79.2)	20 (80.0)	1.000
Diabetes	12 (50.0)	14 (56.0)	0.674
Vascular access, n (%)			0.413
Arteriovenous fistula	22 (91.7)	21 (84.0)	
Arteriovenous graft	2 (8.3)	4 (16.0)	
Dialyzer, n (%)			1.000
FX CorDiax 80	17 (70.8)	18 (72.0)	
FX CorDiax 60	7 (29.2)	7 (28.0)	
Dialysis vintage (mos)	83.6 ± 49.7	70.8 ± 48.4	0.367
Dialysis sessions per week	2.9 ± 0.3	2.9 ± 0.3	0.967
Blood flow rate (mL/min)	245.4 ± 20.8	235.2 ± 19.6	0.084
Dialysate flow rate (mL/min)	500	500	
Dialysis time (min)	238.4 ± 9.2	234.8 ± 12.3	0.259

Abbreviation: MCO, medium cut-off.

### Comparison of laboratory data, ultrafiltration volume, and dialysis adequacy

Table 2 displays the laboratory data, including levels of serum hemoglobin, albumin, calcium, creatinine, phosphate, blood urea nitrogen, total cholesterol, and high-density lipoprotein cholesterol; the ultrafiltration volume; and the single-pool Kt/V; at baseline and 12 weeks after randomization. The MCO and high-flux groups did not show any differences in biochemical markers including serum albumin, and the ultrafiltration volume and dialysis adequacy were also similar at baseline and at 12 weeks.

**Table 2:** Comparisons of the changes in biochemical markers and dialysis adequacy in both dialysis groups.

	Baseline			12 weeks		
	MCO (*n* = 24)	High-flux (*n* = 25)	*P*	MCO (*n* = 24)	High-flux (*n* = 25)	*P*
Hemoglobin (g/dL)	10.6 ± 0.9	10.7 ± 1.1	0.859	10.9 ± 0.9	11.0 ± 1.0	0.697
Albumin (g/dL)	4.11 ± 0.38	4.06 ± 0.33	0.635	3.98 ± 0.27	4.04 ± 0.33	0.450
Creatinine (mg/dL)	10.8 ± 2.5	9.6 ± 2.5	0.093	11.5 ± 2.9	10.0 ± 3.1	0.086
Phosphate (mg/dL)	3.88 ± 0.93	4.05 ± 1.08	0.561	4.08 ± 1.39	4.36 ± 1.35	0.471
BUN (mg/dL)	60.3 ± 16.1	63.2 ± 18.7	0.565	65.5 ± 18.8	60.2 ± 18.2	0.318
Total cholesterol (mg/dL)	125.1 ± 27.7	126.4 ± 28.3	0.874	128.1 ± 31.4	121.6 ± 33.8	0.491
HDL cholesterol (mg/dL)	41.8 ± 12.1	44.1 ± 12.4	0.510	44.7 ± 14.2	43.1 ± 15.7	0.706
Ultrafiltration (L)	1.99 ± 0.84	1.98 ± 0.93	0.964	2.16 ± 0.82	1.98 ± 0.86	0.444
Single-pool Kt/V	1.61 ± 0.22	1.67 ± 0.22	0.296	1.64 ± 0.18	1.68 ± 0.22	0.411

Abbreviations: MCO, medium cut-off; BUN, blood urea nitrogen; HDL, high-density lipoprotein.

### Comparison of middle molecule removal

**Figure 2:**
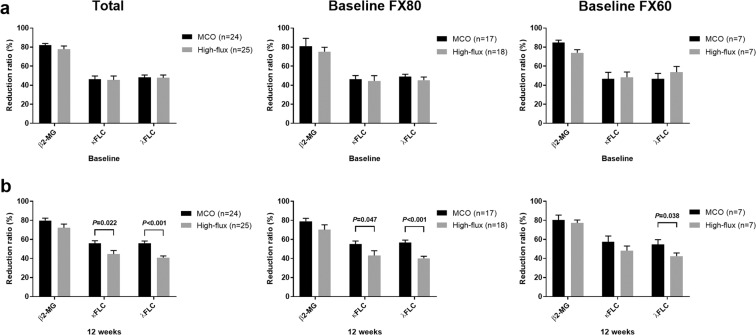
Reduction ratios of middle molecules. (**a**) Reduction ratio at baseline. (**b**) Reduction ratio at 12 weeks after randomization. At baseline, all patients used a high-flux membrane. The error bars indicate the standard error. The specifications of the MCO and high-flux dialyzers are as follows (dialyzer [type of dialyzer, membrane polymer, inner diameter, wall thickness, effective surface area]): Theranova 400 (medium cut-off, polyarylethersulfone-PVP, 180 μm, 35 μm, 1.7 m^2^); FX CorDiax 80 (high-flux, polysulfone-PVP, 185 μm, 35 μm, 1.8 m^2^); FX CorDiax 60 (high-flux, polysulfone-PVP, 185 μm, 35 μm, 1.4 m^2^), respectively. Abbreviations: MCO, medium cut-off; FX80, FX CorDiax 80; FX60, FX CorDiax 60; β2-MG, β2-microglobulin; κFLC, kappa free light chain; λFLC, lambda free light chain; PVP, polyvinylpyrrolidone.

**Figure 3:**
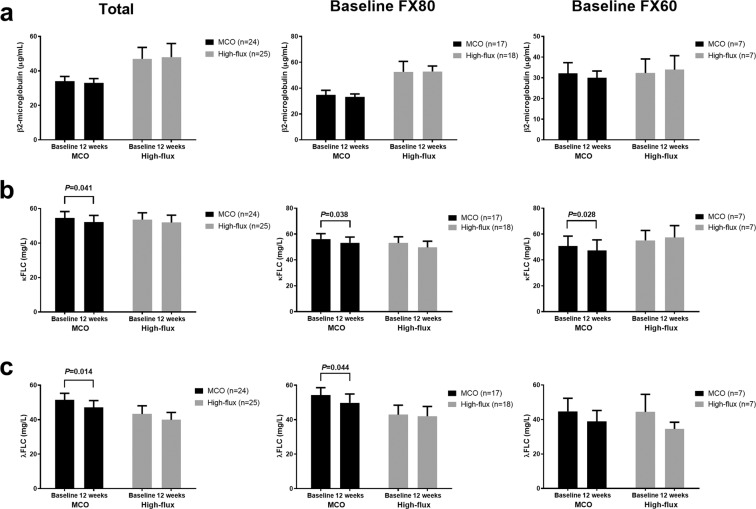
Interval changes in the middle molecules levels. (**a**) β2-microglobulin. (**b**) Kappa free light chain. (**c**) Lambda free light chain. The error bars indicate the standard error. The specification of the MCO and high-flux dialyzers are as follows (dialyzer [type of dialyzer, membrane polymer, inner diameter, wall thickness, effective surface area]): Theranova 400 (medium cut-off, polyarylethersulfone-PVP, 180 μm, 35 μm, 1.7 m^2^); FX CorDiax 80 (high-flux, polysulfone-PVP, 185 μm, 35 μm, 1.8 m^2^); FX CorDiax 60 (high-flux, polysulfone-PVP, 185 μm, 35 μm, 1.4 m^2^), respectively. Abbreviations: MCO, medium cut-off; FX80, FX CorDiax 80; FX60, FX CorDiax 60; κFLC, kappa free light chain; λFLC, lambda free light chain; PVP, polyvinylpyrrolidone.

The serum levels and reduction ratios (RRs) of three middle-sized solutes were evaluated and compared between the groups at baseline and at 12 weeks. The pre-dialysis and post-dialysis levels of the three middle molecules (β2-microglobulin, kappa free light chain [κFLC], and lambda free light chain [λFLC]) did not differ between the MCO and high-flux groups at baseline or at 12 weeks (Table 3). However, the MCO dialyzer displayed better removal of κFLC and λFLC compared with the high-flux dialyzer (κFLC: 55.8 ± 13.7% vs. 44.6 ± 18.9%, *P* = 0.022; λFLC: 56.1 ± 11.4% vs. 40.9 ± 9.0%, *P* < 0.001; Table 4 and Fig. 2). Compared with the patients who used FX CorDiax 80 at baseline, the MCO group also showed a better RR of κFLC and λFLC (both *P* < 0.05), and among patients who used FX CorDiax 60 at baseline, the MCO group showed a better RR of λFLC (*P* = 0.038) (Fig. 2). After 12 weeks of dialysis with an MCO dialyzer, the pre-dialysis levels of κFLC and λFLC were significantly decreased compared to the pre-dialysis levels at baseline (both *P* < 0.05, Fig. 3), but there were no changes in κFLC and λFLC in the high-flux group between baseline and at 12 weeks. Compared with the patients who used FX CorDiax 80 at baseline, the MCO group also showed a significant decrease in the pre-dialysis levels of κFLC and λFLC (both *P* < 0.05), and among patients who used FX CorDiax 60 at baseline, the MCO group showed a significant decrease in the pre-dialysis levels of κFLC (*P* = 0.028) and a decreasing tendency of the pre-dialysis levels of λFLC (*P* = 0.063) (Fig. 3).

**Table 3:** Pre- and post-dialysis middle molecule concentrations in the MCO and high-flux hemodialysis groups.

Baseline	MCO		High-flux		*P* for pre-dialysis	*P* for post-dialysis
	Pre-dialysis	Post-dialysis	Pre-dialysis	Post-dialysis		
β2-microglobulin (µg/mL)	34.0 ± 13.4	6.7 ± 3.8	47.1 ± 31.8	8.3 ± 2.3	0.091	0.106
κFLC (mg/L)	54.6 ± 18.0	35.9 ± 19.0	53.6 ± 19.9	37.3 ± 22.7	0.861	0.822
λFLC (mg/L)	51.5 ± 18.5	30.6 ± 12.5	43.3 ± 23.9	26.9 ± 19.4	0.187	0.434
**12 weeks**	**MCO**		**High-flux**		***P*** **for pre-dialysis**	***P*** **for** **post-dialysis**
	**Pre-dialysis**	**Post-dialysis**	**Pre-dialysis**	**Post-dialysis**		
β2-microglobulin (µg/mL)	32.9 ± 12.4	7.1 ± 4.0	47.9 ± 38.4	15.9 ± 27.8	0.086	0.141
κFLC (mg/L)	52.2 ± 18.7	28.7 ± 17.2	51.9 ± 21.5	35.9 ± 21.8	0.963	0.206
λFLC (mg/L)	47.1 ± 19.4	23.5 ± 9.5	39.9 ± 21.4	28.4 ± 18.3	0.229	0.245

Abbreviations: MCO, medium cut-off; κFLC, kappa free light chain; λFLC, lambda free light chain.

**Table 4:** Reduction ratio of uremic retention solutes.

Reduction ratio (%)	Baseline			12 weeks		
	MCO	High-flux	*P*	MCO	High-flux	*P*
β2-microglobulin	82.1 ± 7.8	77.8 ± 16.2	0.265	79.8 ± 12.2	72.3 ± 18.2	0.109
κFLC	46.5 ± 15.7	45.5 ± 21.0	0.851	55.8 ± 13.7	44.6 ± 18.9	**0.022**
λFLC	48.3 ± 11.6	47.7 ± 14.8	0.865	56.1 ± 11.4	40.9 ± 9.0	**<0.001**

Abbreviations: MCO, medium cut-off; κFLC, kappa free light chain; λFLC, lambda free light chain.

### Comparison of QOL scores

The baseline perceptions of QOL assessed by the Kidney Disease Quality of Life-Short Form (KDQOL-SF) were similar in both groups (Table 5). QOL was reassessed after the 12-week study period. Among the 26 categories included in the QOL questionnaire, the physical function domain score was better in the MCO group than in the high-flux group (75.2 ± 20.8 vs. 59.8 ± 30.1, *P* = 0.042), and the role-physical domain score was also higher in the MCO group (61.5 ± 37.6 vs. 39.0 ± 39.6, *P* = 0.047). The Short Form 36 score tended to be higher in the MCO group than in the high-flux group (61.5 ± 17.7 vs. 51.0 ± 24.1, *P* = 0.088). The kidney disease composite summary (KDCS) and the related detailed item scores did not differ before and after the 12-week treatment in the two groups.

**Table 5:** Quality of life questionnaire scores at baseline and 12 weeks.

	Baseline			12 weeks		
	MCO (*n* = 24)	High-flux (*n* = 25)	*P*	MCO (*n* = 24)	High-flux (*n* = 25)	*P*
Total score	63.7 ± 13.8	57.0 ± 16.4	0.134	63.9 ± 14.4	59.0 ± 17.3	0.283
Kidney disease targeted items	67.9 ± 11.4	62.9 ± 12.3	0.142	66.2 ± 13.3	66.2 ± 12.9	0.995
Symptoms	81.9 ± 13.8	75.4 ± 14.0	0.107	81.3 ± 14.9	78.3 ± 14.6	0.471
Effects of kidney disease	67.6 ± 14.9	60.7 ± 18.9	0.163	65.1 ± 20.3	67.6 ± 18.9	0.654
Burden of kidney disease	40.9 ± 24.4	31.5 ± 26.1	0.200	39.3 ± 27.2	30.8 ± 23.5	0.244
Work status	14.6 ± 27.5	14.0 ± 30.7	0.945	12.5 ± 26.6	18.0 ± 35.0	0.540
Cognitive function	82.5 ± 19.0	83.7 ± 13.6	0.795	78.1 ± 24.1	84.0 ± 17.6	0.328
Quality of social interaction	67.8 ± 18.3	60.5 ± 15.0	0.136	68.1 ± 22.7	67.5 ± 20.3	0.927
Sexual function	57.5 ± 28.8	40.6 ± 42.5	0.500	45.8 ± 35.9	50.0 ± 70.7	0.911
Sleep	64.1 ± 19.3	60.9 ± 17.7	0.553	62.6 ± 15.1	61.6 ± 18.6	0.837
Social support	66.0 ± 22.2	66.0 ± 23.3	0.997	61.8 ± 23.3	73.3 ± 22.1	0.082
Dialysis staff encouragement	87.0 ± 14.0	85.5 ± 16.4	0.736	85.9 ± 15.3	85.5 ± 17.9	0.927
Patient satisfaction	61.8 ± 23.8	60.7 ± 23.0	0.866	61.1 ± 20.1	59.3 ± 22.6	0.773
Short form 36 items	58.9 ± 18.7	50.4 ± 22.6	0.158	61.5 ± 17.7	51.0 ± 24.1	0.088
PCS	61.4 ± 21.7	51.4 ± 25.8	0.150	62.8 ± 20.5	51.7 ± 25.8	0.100
Physical functioning	72.1 ± 23.7	59.4 ± 28.3	0.096	75.2 ± 20.8	59.8 ± 30.1	**0.042**
Role-physical	56.3 ± 39.2	44.0 ± 40.4	0.287	61.5 ± 37.6	39.0 ± 39.6	**0.047**
Pain	70.9 ± 22.9	65.0 ± 28.2	0.424	72.2 ± 24.9	69.3 ± 24.1	0.682
General health	37.9 ± 18.7	36.0 ± 26.0	0.768	35.4 ± 20.1	38.4 ± 27.3	0.666
MCS	55.8 ± 18.1	49.2 ± 21.1	0.249	60.2 ± 16.4	50.5 ± 23.8	0.104
Emotional well-being	54.7 ± 16.0	57.9 ± 18.6	0.515	61.7 ± 16.1	53.4 ± 21.8	0.141
Role-emotional	61.1 ± 40.1	38.7 ± 44.8	0.071	62.5 ± 38.5	45.3 ± 45.0	0.159
Social function	70.3 ± 21.1	62.0 ± 28.1	0.249	69.8 ± 23.6	64.0 ± 26.6	0.425
Energy/fatigue	45.8 ± 20.7	39.8 ± 18.6	0.289	51.7 ± 17.9	43.8 ± 21.6	0.173
Health status compared to one year ago	51.0 ± 21.5	46.0 ± 25.7	0.461	53.1 ± 23.7	46.0 ± 24.7	0.308
Overall health rate	57.9 ± 22.1	56.4 ± 25.2	0.824	58.8 ± 22.5	50.0 ± 26.3	0.218

Values are shown as the mean ± standard deviation.

Abbreviations: PCS, physical composite summary; MCS, mental composite summary.

### Comparison of pruritus scores

The clinical characteristics of uremic pruritus at baseline and after 12 weeks of the study treatment are shown (Table 6). At baseline, the morning pruritus intensity was worse in the MCO group than in the high-flux group (1.92 ± 1.06 vs. 1.40 ± 0.50, *P* = 0.033); however, this difference was not observed after 12 weeks (1.54 ± 0.72 vs. 1.64 ± 0.86, *P* = 0.667). The pruritus distribution in the morning was smaller in the MCO group after 12 weeks (1.29 ± 0.46 vs. 1.64 ± 0.64, *P* = 0.034). The MCO group also experienced less frequent sleep disturbances caused by pruritus-related scratching (0.25 ± 0.53 vs. 1.00 ± 1.47, *P* = 0.023). The visual analog scale (VAS) scores tended to be higher in the MCO group, but the differences were not significant.

**Table 6:** Assessment of uremic pruritus at baseline and 12 weeks.

	Baseline			12 weeks		
	MCO (*n* = 24)	High-flux (*n* = 25)	*P*	MCO (*n* = 24)	High-flux (*n* = 25)	*P*
**Severity**						
Morning	1.92 ± 1.06	1.40 ± 0.50	**0.033**	1.54 ± 0.72	1.64 ± 0.86	0.667
Afternoon	2.00 ± 1.14	1.72 ± 0.84	0.332	1.88 ± 0.95	1.84 ± 1.07	0.904
**Distribution**						
Morning	1.42 ± 0.58	1.48 ± 0.71	0.736	1.29 ± 0.46	1.64 ± 0.64	**0.034**
Afternoon	1.46 ± 0.59	1.56 ± 0.96	0.659	1.38 ± 0.65	1.56 ± 0.71	0.347
**Sleep disturbance**						
Frequency of waking from sleep	0.83 ± 1.05	0.68 ± 1.28	0.650	0.75 ± 0.85	1.32 ± 1.60	0.126
Frequency of scratching during sleep	0.38 ± 0.92	0.24 ± 0.72	0.571	0.25 ± 0.53	1.00 ± 1.47	**0.023**
Total score by measuring system	8.58 ± 7.74	7.20 ± 7.58	0.530	6.92 ± 5.98	9.92 ± 8.23	0.152
**VAS scoring system**						
Morning	2.58 ± 2.24	2.14 ± 2.28	0.496	2.50 ± 1.93	3.34 ± 2.82	0.232
Afternoon	3.04 ± 2.57	2.74 ± 2.53	0.680	3.46 ± 2.32	4.24 ± 3.18	0.333
Average	2.81 ± 2.19	2.44 ± 2.31	0.565	2.98 ± 1.98	3.79 ± 2.91	0.262

Abbreviations: MCO, medium cut-off; VAS, visual analog scale.

### Correlation of dialyzer to quality of life scores and to pruritus scores

Table 7 shows the factors associated with the physical component domains of the QOL questionnaire and pruritus status at 12 weeks. In the multivariate linear regression analysis, the MCO dialyzer displayed positive associations with physical component domains such as physical functioning and physical role at 12 weeks after randomization (physical functioning: unstandardized regression coefficient [B], 16.67 [95% confidence interval (CI), 3.44, 29.90], *P* = 0.015; role-physical: B, 25.32 [95% CI, 3.43, 47.20], *P* = 0.024). In addition, the MCO dialyzer showed an inverse correlation with the morning pruritus distribution and frequency of scratching during sleep (morning pruritus distribution: B, − 0.40 [95% CI, − 0.73, −0.06], *P* = 0.022; frequency of scratching during sleep: B, − 0.92 [95% CI, − 1.57, −0.28], *P* = 0.006).

**Table 7:** Multivariate linear regression analysis of factors associated with physical component domains on quality of life questionnaires and pruritus status at 12 weeks.

	Physical functioning			Role-physical			Pruritus distribution, morning			Sleep disturbance, Frequency of scratching during sleep		
	B (95% CI)	β	*P*	B (95% CI)	β	*P*	B (95% CI)	β	*P*	B (95% CI)	β	*P*
Dialyzer type	16.67 (3.44, 29.90)	0.31	**0.015**	25.32 (3.43, 47.20)	0.32	**0.024**	−0.40 (−0.73, −0.06)	−0.34	**0.022**	−0.92 (−1.57, −0.28)	−0.40	**0.006**
Age	−0.17 (−0.68, 0.33)	−0.09	0.495	−0.24 (−1.08, 0.59)	0.56	0.560	0.01 (−0.01, 0.02)	0.17	0.274	−0.02 (−0.04, 0.01)	−0.21	0.173
Gender	−3.79 (−20.30, 12.72)	−0.07	0.645	8.00 (−19.31, 35.30)	0.56	0.557	−0.25 (−0.66, 0.17)	−0.20	0.242	−0.46 (−1.26, 0.35)	−0.19	0.256
Diabetes	−14.33 (−27.81, −0.86)	−0.27	**0.038**	−26.66 (−48.95, −4.38)	−0.34	**0.020**	0.07 (−0.27, 0.41)	0.06	0.681	−0.33 (−0.32, 0.99)	0.14	0.313
Hemoglobin	−2.44 (−10.08, 5.21)	−0.08	0.523	−2.99 (−15.63, 9.66)	−0.07	0.636	0.15 (−0.05, 0.34)	0.23	0.135	0.21 (−0.17, 0.58)	0.16	0.269
Albumin	40.43 (15.58, 65.27)	0.45	**0.002**	28.44 (−12.65, 69.53)	0.21	0.170	−0.32 (−0.95, 0.31)	−0.16	0.307	−1.65 (−2.85, −0.44)	−0.42	**0.009**
Total cholesterol	−0.09 (−0.35, 0.16)	−0.11	0.458	−0.33 (−0.75, 0.09)	−0.27	0.088	0.003 (−0.003, 0.010)	0.18	0.310	0.002 (−0.011, 0.014)	0.04	0.805

Adjusted *R*-squared is 0.343 in the physical functioning domain, 0.186 in the role-physical domain, 0.100 for the morning pruritus distribution, and 0.178 for the frequency of scratching during sleep. The reference dialyzer is a high-flux dialyzer, and the reference gender is male.

Abbreviations: B, unstandardized regression coefficient; CI, confidence interval; β, standardized coefficient.

### Adverse events

Throughout the 12 weeks of the study, there were no serious adverse events including cardiovascular events, patient death, or a decline of blood pressure that required dialyzer changes.

## Discussion

This randomized controlled trial demonstrated the beneficial effects of MCO dialyzers on patient-reported outcomes in chronic HD patients. Patients in the MCO group had better KDQOL-SF physical domain scores than patients in the high-flux group. Our results indicate that HD with an MCO dialyzer reduced the distribution and sleep disturbances caused by uremic pruritus better than HD with a high-flux dialyzer. We speculate that the improvement in the physical domains of QOL was due to the more effective removal of middle molecules. This is the first randomized controlled study to evaluate the effects of a new MCO dialyzer on the QOL of HD patients.

Previous studies have identified the detrimental effects of middle-sized solutes on various aspects of health in patients with ESRD, including QOL^21^. Although researchers have attempted to identify associations between middle molecule clearance and QOL, studies that compared the effects of the dialysis method or dialyzer on QOL have not shown consistent results. Some studies have shown that patients on HDF had better QOL than patients on conventional HD^22,23^, but other studies showed no difference in QOL between HDF and HD groups^10,24,25^. Among the various domains of QOL, the physical domains of maintenance HD patients have been reported to decline over time, whereas the mental and emotional domains remained relatively stable^26,27^. An observational study also showed that physical components, in particular, could be improved after HD with a different dialysis method;^22^ this study reported that the differences in QOL questionnaire scores based on the dialysis methods (HDF vs. high-flux or low-flux HD) were most pronounced in physical domains. Our results were consistent with previous studies showing that HD with an MCO dialyzer was associated with higher physical functioning and role-physical scores on the KDQOL-SF physical composite summary (PCS). The effect of the MCO dialyzer on QOL is likely related to the better removal rate of middle molecules compared to high-flux dialyzers. The improvements in the physical components of the QOL questionnaire over a relatively short exposure period (12 weeks) of MCO dialyzer use are attributed to improvements in the removal rates of middle molecules that occurred concurrently with the change of the dialyzer. Previous studies that compared QOL between HDF and conventional HD have also shown improvements in QOL in short periods of one month and three months after changing to HDF, which may have been caused by increased convection^23,28^. In addition, confounding factors are unlikely because the baseline characteristics of the MCO and high-flux groups did not differ, and the effect of the MCO dialyzer on the physical component domains remained significant after the linear regression analysis. Further long-term studies of the sustainability of QOL improvement are necessary in the near future.

A previous large study showed that approximately 42% of patients with HD experience moderate to extreme pruritus^29^. Severe uremic pruritus may cause deteriorating QOL and poor clinical outcomes in chronic HD patients^30,31^. Reasons for the occurrence of pruritus remain unknown, but the possibility of an association of uremic pruritus with middle molecules such as β2-microglobulin has been reported; therefore, the increased clearance of middle molecules may help reduce uremic pruritus^32,33^. Goeksel *et al*. also reported that the improved removal of high-molecular weight uremic retention solutes using a highly permeable polymethylmethacrylate dialyzer with absorption capacity improves uremic pruritus^34^. At baseline, uremic pruritus was worse in the MCO group, but after 12 weeks of HD with an MCO dialyzer, uremic pruritus status improved more in the MCO group than in the high-flux group. However, there is a limit to the generalizability of the results because many patients had mild to moderate pruritus. Further study is necessary to determine whether this effect is maintained long-term and whether it is effective in patients with severe pruritus.

To accurately compare the difference in the clearance of middle molecules according to size between the different dialyzers, we tested the removal rates of three middle molecules according to MW. Among them, β2-microglobulin, a small middle molecule, manifested similar rates of removal; however, larger middle molecules such as κFLC and λFLC had higher rates of removal with the MCO dialyzer. This finding agrees with the results of a previous study reported by Kirsch *et al*., which has been cited in the data sheet of the MCO dialyzer manufacturer; this study shows a greater RR of a wide range of middle molecules including β2-microglobulin, κFLC, and λFLC with the MCO dialyzer than with the high-flux dialyzer^35^. These investigators also reported that small solute clearance did not differ between the MCO and high-flux groups. Compared with the results of Kirsch *et al*., the RRs obtained in our study were slightly lower, and the RRs of smaller middle molecules (β2-microglobulin) did not differ compared to that of high-flux HD. This difference might be attributable to the lower blood flow rates (shown in Table 1) usually applied to Asian dialysis populations and the lower mean dry weight in our subjects compared with those of Kirsch *et al*. Although directly comparing the RRs of specific molecules to the previous study is difficult, the overall removal of larger middle molecules by the MCO dialyzer was superior than that of the high-flux dialyzer. Our study suggests that the MCO dialyzer offers an opportunity for the better removal of larger middle molecules in maintenance HD patients than conventional high-flux dialyzers, and long-term studies are necessary to clearly determine the effects on serum concentration.

Given that larger middle molecule clearance was only achieved by using new dialyzers with a modified sieving profile, this technology, called expanded HD^36^, could be used in patients who are intolerant of high convection volume for HDF or in centers without the infrastructures and specialized monitors for HDF. According to the multi-dimensional membrane classification, various factors can be used to evaluate the dialyzer class, such as composition, sieving for middle molecules, molecular weight retention onset (MWRO) for different MW solutes, fiber diameter, and thickness^36^. Thus, the MCO dialyzer has a smaller surface area than conventional high-flux dialyzers, but it can obtain greater convection and shows the effective removal of middle molecules. Although the MCO dialyzer was not compared with HDF in our study, we speculate that use of the MCO dialyzer could improve QOL in patients treated with HDF because the MCO dialyzer has displayed better removal of larger middle molecules than HDF^35,37^.

One of the major concerns of the MCO dialyzer is the risk of albumin loss through the more permeable membrane. Kirsch *et al*. reported that the MCO dialyzer removed more albumin than high-flux HD and HDF^35^. By contrast, Garcia-Prieto *et al*. showed that albumin loss was much lower with an MCO dialyzer compared to online HDF^38^. Although the mean ranges of albumin loss in the two studies ranged widely from 0.03 to 4.9 g/session, the amounts were comparable with the range reported for HDF with high-flux dialyzers^39–41^. In several randomized studies on HDF, this amount of albumin removal did not result in any significant change in serum albumin levels in the patients treated with HDF^12–15^. In our study, the serum albumin concentration after three months of using the MCO dialyzer decreased by a mean of 0.13 ± 0.23 mg/dL from baseline; however, the serum albumin concentrations did not differ between the two groups. This suggests that HD with an MCO dialyzer might initially cause a small but non-significant decrease in serum albumin levels. Further studies should investigate whether the decrease could be compensated for or could affect serum albumin concentrations after longer use of an MCO dialyzer.

This study has several limitations. First, the sample size was small, and the study duration was insufficient to evaluate definite effects of the MCO membrane. Nevertheless, we demonstrated significant interval changes in QOL and pruritus after treatment with an MCO dialyzer. Our results of an initial period of MCO dialyzer use provide information about adverse events and changes in laboratory findings that may be helpful for identifying candidates for novel therapies. Second, the Theranova 500 dialyzer, which has a greater surface area (2.0 m^2^) than the Theranova 400 dialyzer (1.7 m^2^), was not applied in the MCO group because the Theranova 500 has not yet been introduced in Korea; however, it has been reported that the surface area of the MCO dialyzer was not correlated with depurative effectiveness in middle molecules^42^. Third, we could not estimate the actual extent of solute removal or prove the exact pathophysiologic correlations between middle molecules and the physical components of QOL and uremic pruritus. However, the strengths of our study include that it is the first randomized controlled prospective trial comparing the QOL of maintenance HD patients treated with MCO and high-flux dialyzers. This study will lead to larger and longer subsequent studies involving HDF, which is a more convective method than high-flux dialysis.

In conclusion, compared to a high-flux dialyzer, the new MCO dialyzer may improve self-reported outcomes, particularly the physical domains of QOL and uremic pruritus, in stable maintenance HD patients who use permanent dialysis access. The MCO dialyzer also had a non-significant effect on the serum albumin concentration over 12 weeks of treatment.

## Methods

### Study design

This study is a randomized, prospective, controlled, open-label, phase 4 trial in patients receiving maintenance HD. Patients treated with maintenance HD at the Kyungpook National University Hospital were enrolled and randomized starting in July 2018, and the study was completed in January 2019. This study was conducted in accordance with the ethical principles of the Declaration of Helsinki, and the Institutional Review Board of Kyungpook National University Hospital approved the study protocol (KNUH 2017-11-024). This study was registered with the Clinical Research Information Service (CRiS) at the Korea Centers for Disease Control and Prevention (KCT0003026; registration date: 25/07/2018).

The detailed inclusion and exclusion criteria are provided at CRiS (http://cris.nih.go.kr). Briefly, a total of 50 patients receiving maintenance high-flux membrane HD for more than three months were enrolled. Other inclusion criteria were as follows: older than 18 years, vascular access by arteriovenous fistula/graft, adequate dialysis (Kt/V > 1.2), and agreement to participate in the clinical study. The exclusion criteria were as follows: low-flux HD, vascular access for dialysis by temporary catheter, scheduled kidney transplantation, admission within three months, hematologic malignancy, monoclonal gammopathy, solid organ malignancy, active infection, human immunodeficiency virus infection, and enrollment in other clinical trials within three months. All patients provided written informed consent.

After enrollment, all patients were randomly divided into MCO and high-flux groups at a 1:1 ratio according to the random number table method with a random number table provided by a statistician who was not involved in the study. The random assignment results were immediately reported to the participants and physicians. The patients and physicians were unblinded to the assignment. The MCO group patients switched from a high-flux membrane (FX CorDiax 80 or 60; Fresenius Medical Care Deutschland, Bad Homburg, Germany) to a Theranova 400 dialyzer (Baxter International Inc., Hechingen, Germany), and the high-flux group continued HD with a high-flux membrane. The detailed characteristics of the dialyzers are listed in Supplementary Table S1 and are described briefly as follows (dialyzer [membrane polymer, inner diameter, wall thickness, effective surface area]): Theranova 400 (polyarylethersulfone-polyvinylpyrrolidone [PVP], 180 μm, 35 μm, 1.7 m^2^); FX CorDiax 80 (polysulfone-PVP, 185 μm, 35 μm, 1.8 m^2^); FX CorDiax 60 (polysulfone-PVP, 185 μm, 35 μm, 1.4 m^2^), respectively. The dialysis treatment details including dialysis time per session, dialysis frequency per week, blood flow, and dialysate flow did not change during the study period. The recommended dialysis prescriptions are as follows: dialysis time: 4 hours, dialysate flow rate: 500 mL/min, blood flow rate: 200–300 mL/min, and target Kt/V > 1.2. Basic demographics, dialysis information including dialysis adequacy, and biochemical data were collected at baseline. After 12 weeks of treatment, dialysis information, and biochemical data were reassessed. Questionnaires about QOL and pruritus were completed at baseline and at 12 weeks. Blood samples to identify middle molecule levels were obtained before and at the end of dialysis.

### Data collection and analysis

#### QOL

The patients completed the KDQOL-SF^43^ questionnaire at the beginning of the treatment period and after 12 weeks of treatment. The KDQOL-SF consists of 80 detailed items in 19 domains. The KDCS category was the sum of kidney disease targeted items. The Short Form 36 was classified into two groups: a PCS that reflects the patients’ physical condition, pain, and general health status; and a mental composite summary that reflects the patients’ emotional and social condition.

#### Uremic pruritus

Uremic pruritus was assessed using the scoring system modified by Pauli-Magnus. The pruritus scoring questionnaire was composed of severity, distribution, and sleep disturbance categories^44^.

Severity. Scores of 1, 2, 4, and 5 points were recorded for a slight itchy sensation without scratching, the need to scratch without excoriations, scratching with excoriation, and pruritus with total restlessness, respectively.

Distribution. Itching at less than two sites, more than two sites, and generalized itching received 1, 2, and 3 points, respectively. The scores of severity and distribution were assessed in the morning and the afternoon. The two scores were multiplied separately to achieve a maximum of 30 points.

Sleep disturbance. Each waking episode from itching received 2 points (maximum 10 points). Each nighttime scratching episode causing excoriations received 1 point (maximum 5 points). The final score was calculated as the addition of the sleep disturbance score and the severity-distribution product (maximum of 45 points).

The VAS was used to evaluate the subjective intensity of pruritus^45^. The patients reported their itching intensity on a 10-point VAS with 0 indicating no pruritus and 10 indicating unbearable pruritus.

### Sampling and analysis

Blood samples were collected before and after the dialysis sessions at baseline and 12 weeks after the treatment. Post-dialysis samples were obtained using the slow-flow method^46^. At the time of the baseline examination, all patients underwent HD using a high-flux membrane dialyzer. The samples were collected in tubes containing a serum-separating agent and then centrifuged for 10 minutes at 3,000 rpm and 4 °C. Then, the serum samples were immediately frozen and stored at −80 °C until analysis. The middle molecule concentrations were measured using commercially available ELISA kits: β2-microglobulin (MW, 11.8 kDa) with the Beta-2 Microglobulin Human SimpleStep ELISA kit (Abcam, Cambridge, UK) and κFLC (monomeric MW, 22.5 kDa) and λFLC (dimeric MW, 45.0 kDa) with the Human Immunoglobulin Free Light Chains Kappa and Lambda ELISA kit (BioVendor-Laboratorni medicina a.s., Brno, Czech Republic). All assays were performed in accordance with the manufacturer’s instructions.

#### Calculations

The RRs of each middle molecule were calculated based on the pre- and post-dialysis plasma concentrations of the corresponding molecules. To compensate for hemoconcentration during HD, the RRs of the middle molecules were calculated using the Bergstrom and Wehle formula^47^. RR was calculated as:

$${\rm{R}}{\rm{R}}({\rm{ \% }})=(1\text{-}{{\rm{C}}}_{{\rm{P}}{\rm{o}}{\rm{s}}{\rm{t}}}/{{\rm{C}}}_{{\rm{P}}{\rm{r}}{\rm{e}}})\times 100$$

in which C_Pre_ and C_Post_ are the measured plasma concentrations of the molecule before and at the end of HD, respectively. C_Post_ was further corrected for ultrafiltration (C_Post-corr_) as follows:

$${{\rm{C}}}_{{\rm{P}}{\rm{o}}{\rm{s}}{\rm{t}}-{\rm{c}}{\rm{o}}{\rm{r}}{\rm{r}}}={{\rm{C}}}_{{\rm{P}}{\rm{o}}{\rm{s}}{\rm{t}}}/[1+({{\rm{B}}{\rm{W}}}_{{\rm{P}}{\rm{r}}{\rm{e}}}\text{-}{{\rm{B}}{\rm{W}}}_{{\rm{P}}{\rm{o}}{\rm{s}}{\rm{t}}})/(0.2\times {{\rm{B}}{\rm{W}}}_{{\rm{P}}{\rm{o}}{\rm{s}}{\rm{t}}})]$$

in which BW_Pre_ and BW_Post_ are the patient body weight before and at the end of HD, respectively.

#### Study outcomes

The primary outcome was the KDQOL-SF and pruritus assessment, which were evaluated using the questionnaire and VAS, respectively. The secondary outcomes were pre-dialysis plasma concentrations and RRs of middle molecules assessed at baseline and 12 weeks after randomization, which were regarded as factors potentially associated with QOL and pruritus.

### Statistical analysis

There have been no previous studies on the effect of the MCO membrane on QOL, but we determined the sample size based on the results of a previous study that compared QOL among patients undergoing HDF, high-flux HD, and low-flux HD^17^. For the primary endpoint, we assumed that the power would be 80% with a two-sided type 1 error rate of 5%, and the standard deviation for the difference in QOL would be 15. We also predicted a 10% dropout rate for both groups. Thus, to identify a minimum difference of 12 in the subparameter scores of the QOL questionnaire, 25 patients were required in each the MCO and high-flux groups. All patients who completed the questionnaires at least once after enrollment were included in the analysis. All variables were expressed as means ± SD or number (percentage, %), depending on the nature of the variables. The Student’s *t*-test was applied to determine differences between continuous variables, and Pearson’s chi-square test or Fisher’s exact test was used to determine the differences in categorical variables. A multiple linear regression analysis was used to identify the factors associated with physical component domains on the QOL questionnaires. The statistical analyses were performed with SPSS version 20.0 (SPSS, Chicago, IL, USA). A *P* value of less than 0.05 was considered statistically significant.

## Supplementary information

Supplementary information.

## Data Availability

The datasets generated during and/or analyzed during the current study are available from the corresponding author on reasonable request.
