# Supplementary material for: Randomized controlled trial of medium cut-off versus high-flux dialyzers on quality of life outcomes in maintenance hemodialysis patients
Source: Sci Rep. 2020 May 8;10:7780. doi: 10.1038/s41598-020-64622-z (PMC7210312; doi:10.1038/s41598-020-64622-z)
Supplement: Supplementary file 1 — Supplementary information. [file 41598_2020_64622_MOESM1_ESM.pdf]

Randomized controlled trial of medium cut-off versus high-flux dialyzers on quality of life outcomes in maintenance hemodialysis patients

Jeong-Hoon Lim[1], Yeongwoo Park[2], Ju-Min Yook[1], Soon-Youn Choi[1], Hee-Yeon Jung[1], Ji-Young Choi[1], Sun-Hee Park[1], Chan-Duck Kim[1], Yong-Lim Kim[1] and Jang-Hee Cho[1 *]

[1]Department of Internal Medicine, School of Medicine, Kyungpook National University, Kyungpook National University Hospital, Daegu, South Korea. [2]Department of Statistics, Kyungpook National University, Daegu, South Korea

Supplementary Information Listing:

Supplementary Table S1

[*]Corresponding author:

Jang-Hee Cho, MD, PhD

Associate Professor

Division of Nephrology, Department of Internal Medicine

Kyungpook National University Hospital, 130 Dongdeok-ro, Jung-gu, Daegu, 41944, Korea

Tel: +82-53-200-5550

Fax: +82-53-426-2046

E-mail: jh-cho@knu.ac.k

Supplementary Table S1. Characteristics of dialyzers

Dialyzer	Membrane polymer	Membrane type	Inner diameter (μm)	Wall thickness (μm)	Effective surface area (m^2)	Ultrafiltration coefficient (mL/h/mmHg)	Priming volume (mL)	Sterilization
Therenova 400	Polyarylethersulfone-polyvinylpyrrolidone	Medium cut-off	180	35	1.7	48	91	Steam
FX CorDiax 80	Polysulfone-polyvinylpyrrolidone	High-flux	185	35	1.8	64	95	Steam
FX CorDiax 60	Polysulfone-polyvinylpyrrolidone	High-flux	185	35	1.4	47	74	Steam
